# Time to first passage of meconium and defecation frequency preceding necrotizing enterocolitis in preterm infants: a case–control study

**DOI:** 10.1007/s00431-023-05035-8

**Published:** 2023-06-22

**Authors:** Nancy Deianova, Quincy Moonen, Sientje E. Sluis, Hendrik J. Niemarkt, Wouter J. de Jonge, Marc A. Benninga, Nanne K. H. de Boer, Helen L. Tanger, Mirjam M. van Weissenbruch, Anton H. van Kaam, Tim G. J. de Meij, Ilan J. N. Koppen

**Affiliations:** 1 Department of Pediatric Gastroenterology, Emma Children’s Hospital, Amsterdam Gastroenterology Endocrinology Metabolism Research Institute, Amsterdam UMC, Location AMC, Amsterdam, the Netherlands; 2 Department of Pediatric Gastroenterology, Amsterdam UMC Location University of Amsterdam. Amsterdam Reproduction & Development Research Institute, Amsterdam, the Netherlands; 3 Department of Neonatology, Máxima Medical Center, Veldhoven, the Netherlands; 4 Tytgat Institute for Liver and Intestinal Research, Amsterdam Gastroenterology Endocrinology Metabolism Research Institute, University of Amsterdam, Amsterdam UMC, Amsterdam, the Netherlands; 5 Department of Gastroenterology and Hepatology, Amsterdam UMC, Amsterdam Gastroenterology Endocrinology Metabolism Research Institute, Amsterdam, the Netherlands; 6 Department of Pediatrics, Haaglanden Medical Center, the Hague, the Netherlands; 7 Department of Neonatology, Emma Children’s Hospital Amsterdam Reproduction and Development Research Institute, Amsterdam, the Netherlands

**Keywords:** Necrotizing enterocolitis, Preterm, Meconium passage, Defecation frequency, Dysmotility

## Abstract

Necrotizing enterocolitis (NEC) is associated with significant morbidity and mortality in preterm infants. Early recognition and treatment of NEC are critical to improving outcomes. Enteric nervous system (ENS) immaturity has been proposed as a key factor in NEC pathophysiology. Gastrointestinal dysmotility is associated with ENS immaturity and may serve as a predictive factor for the development of NEC. In this case–control study, preterm infants (gestational age (GA) < 30 weeks) were included in two level-IV neonatal intensive care units. Infants with NEC in the first month of life were 1:3 matched to controls based on GA (± 3 days). Odds ratios for NEC development were analyzed by logistic regression for time to first passage of meconium (TFPM), duration of meconial stool, and mean daily defecation frequency over the 72 h preceding clinical NEC onset (DF < T0). A total of 39 NEC cases and 117 matched controls (median GA 27 + 4 weeks) were included. Median TFPM was comparable in cases and controls (36 h [IQR 13–65] *vs.* 30 h [IQR 9–66], p = 0.83). In 21% of both cases and controls, TFPM was ≥ 72 h (p = 0.87). Duration of meconial stool and DF < T0 were comparable in the NEC and control group (median 4 and 3, resp. in both groups). Odds of NEC were not significantly associated with TFPM, duration of meconial stools, and DF < T0 (adjusted odds ratio [95% confidence interval]: 1.00 [0.99–1.03], 1.16 [0.86–1.55] and 0.97 [0.72–1.31], resp.).

*Conclusion*: In this cohort, no association was found between TFPM, duration of meconium stool, and DF < T0 and the development of NEC.

**Table:** 

**What is Known:** *• Necrotizing enterocolitis (NEC) is a life-threatening acute intestinal inflammatory disease of the young preterm infant. Early clinical risk factors for NEC have been investigated in order to facilitate early diagnosis and treatment.* • *Signs of disrupted gastrointestinal mobility, such as gastric retention and paralytic ileus, have been established to support the diagnosis of NEC. Nevertheless, defecation patterns have insufficiently been studied in relation to the disease.*	
**What is New:** *• Defecation patterns in the three days preceding NEC did not differ from gestational age-matched controls of corresponding postnatal age. Additionally, the first passage of meconium and the duration of meconium passage were comparable between cases and controls. Currently, defecation patterns are not useful as early warning signs for NEC. It remains to be elucidated whether these parameters are different based on the location of intestinal necrosis.*

**Supplementary Information:**

The online version contains supplementary material available at 10.1007/s00431-023-05035-8.

## Introduction

Necrotizing enterocolitis (NEC) is an important cause of morbidity and mortality in premature infants [1–4]. In very low birth weight infants (≤ 1500 g), the prevalence rate of NEC is estimated at approximately 5–7% with high mortality rates varying from 20–50% [5]. The pathophysiology of NEC remains incompletely understood and is considered to be multifactorial. Historically, a pathophysiological triad is described, including intestinal ischemia, enteral nutrition, and the presence of pathogenic organisms in the gut [6].

The diagnosis and staging of NEC primarily rely on a combination of clinical signs and laboratory and radiological findings, incorporated in the modified Bell’s staging criteria [7]. Timely recognition of NEC is challenging because the clinical presentation is often subtle and it has an overlap with other diseases such as sepsis and spontaneous intestinal perforation (SIP) [3, 8]. To address this challenge, several clinical and biomarker-based risk models have been developed [9, 10]. However, to date, these have not led to substantial improvement in diagnostic accuracy nor better treatment outcomes [3, 11].

Symptoms related to defecation patterns have often been overlooked in risk factor assessment of NEC, despite other signs of impaired gastrointestinal motility, such as gastric retention, being associated with the development of this condition [7, 12]. Intestinal motility might be impaired due to immaturity of the enteric nervous system (ENS), characterized by low numbers of neural and glial cells. This can contribute to slowing down peristalsis, potentially promoting bacterial overgrowth and dysbiosis, which in turn is associated with mucosal injury and inflammation characteristic of NEC [12–15].

Current evidence on defecation patterns preceding NEC is limited. Both delayed time to first passage of meconium (TFPM) and irregular defecation frequency have been associated with disorganization and immaturity of the ENS [16, 17]. Available literature consists of small observational studies, some including unconfirmed cases of NEC [7, 18–20]. Additionally, studies are hampered by confounding factors between cases and controls [7, 18–20].

Therefore, we aimed to assess the relationship between TFPM, duration of meconium production, and mean defecation frequency in the three days preceding NEC (DF < T0), in preterm neonates (< 30 weeks of gestation). We hypothesized that signs of gut dysmotility may become apparent early in life in infants who later develop NEC, resulting in a delayed TFPM, prolonged production of meconium, and a decreased DF < T0. All three parameters were evaluated as potential early clinical warning signs.

## Methods

### Study design

This study was embedded in an ongoing prospective multicenter cohort study with the primary objective of identifying novel non-invasive biomarkers for late-onset sepsis (LOS) and NEC in preterm-born infants [21].

### Subjects

Infants born before 30 weeks of gestation who were admitted to Amsterdam UMC (locations VUmc and AMC, Amsterdam, the Netherlands) between December 2015 and January 2020 were eligible for inclusion. Subjects were excluded in case of congenital gastrointestinal diseases (e.g. anal atresia, duodenal atresia, Hirschsprung disease), a meconium plug with blow-out ileum, development of sepsis unrelated to NEC up to 28 days postnatal (as defined by the *Vermont Oxford Network* [22]), meningitis, SIP, and/or abdominal surgery unrelated to NEC. Sepsis-related to NEC was defined as sepsis within three days before NEC onset and was not excluded.

NEC was classified and diagnosed according to the modified Bell’s criteria [7]. All infants suspected of, or diagnosed with, NEC by their treating clinician were retrospectively reviewed and staged by two clinical experts (HN, TdM). Neither were involved in the clinical care of the children. Only confirmed cases of NEC, Bell’s stage IIa or higher, were included [7]. In case of discrepancy, cases were re-evaluated by the experts until a consensus was reached.

Each case of NEC was matched to three controls, based on gestational age (GA) in days with a range of three days. NEC subjects that could not be matched to three controls were excluded.

### Data collection

Baseline demographic and clinical variables were collected from the electronic patient record. Enteral feeding type was defined as human milk if > 80% of enteral feeding consisted of maternal or donor milk. The enteral feeding type was first determined as either formula or human milk for each of the days before the onset of NEC or the corresponding postnatal age of the controls (T_0_). If the infant was predominantly fed with human milk more than 80% of the days, we categorized the neonate as human milk-fed and in the other cases, as formula-fed. The cut-off is arbitrary and has been applied in our previous studies [23]. It is applied to avoid that infants who received formula milk as an exception, e.g. due to delayed preparation of the human milk, would directly be categorized as formula-fed. No probiotics were administered during the study period.

The number of days of parenteral feeding was defined as the days that > 30 ml/kg/day of parenteral nutrition was administered before T_0_. The age of full enteral feeding was defined as the age at which enteral feeding was ≥ 120 ml/kg/day. The TFPM was retrospectively collected from the electronic medical charts based on nurse reports. Therefore, TFPM was collected with the precision ± 3 h, as diaper change was planned every 3 h. Infants with meconium-stained amniotic fluid were categorized into the subgroup of infants who passed meconium immediately after birth. Duration of meconium passage until first transitional stool was reported per day. The transitional stool was defined as brown, green, or yellowish pasty stools. The collection of data on the administration of enemas was based on nurse reports. Enemas were administered based on the caregiver’s judgement. A local protocol for delayed passage of meconium, based on symptoms (e.g. gastric retention, regurgitation, feeding intolerance, abdominal distension) and symptom severity, was designed and was gradually implemented after January 2020. The median DF < T0 was calculated as the average number of stools per day during the three days preceding NEC diagnosis or the corresponding postnatal ages of controls.

### Ethical approval

The study was approved by the local Medical Ethical Review Boards (protocol number A2020.190) of both participating centers and written informed consent was obtained from the parents or legal caretakers of included infants.

### Statistical analyses

For the statistical analyses of clinical and demographical data, Statistical Package for the Social Sciences (SPSS^®^) (version 26.0, IBM^®^, New York City) was used. The distribution of the data was assessed by the shape of the histogram and in case of uncertainty, the Shapiro–Wilk test was applied. Parametric, continuous data were described as mean and standard deviation (SD), and comparisons between groups were performed using the independent samples Student’s t-test. Non-parametric, continuous data were described as median and interquartile range (IQR), and groups were compared using the Mann–Whitney U test. For comparison of categorical data, described as numbers and percentages, Pearson’s Chi-squared test or Fisher’s exact tests were used. A p-value of < 0.05 was deemed statistically significant.

The TFPM was analyzed both as a categorical and continuous variable. First, four TFPM categories (< 24 h, 24–48, 48-72 h, and ≥ 72 h) were assessed in relation to NEC and gestational age, by applying the Chi-squared test. Then, associations between NEC and TFPM, number of days of meconium passage (from first passage of meconium to first transitional stool), and DF < T0 were analyzed by univariate and multivariate logistic regression methods, all as continuous variables.

In the multivariate models, odds ratios (OR) were adjusted for potential clinical and demographic confounding variables. Confounders were only selected if they were correlated to the outcome (Pearson correlation test p-value < 0.05) and changed the regression coefficient by more than 10% compared to the univariate regression coefficient. To avoid overfitting, the number of allowed confounders was 10% of the smallest group. Results from the logistic regression were reported as OR and adjusted OR (aOR), along with the respective 95% confidence interval (95%CI).

A post-hoc analysis was performed to analyze TFPM, number of days of meconium passage and DF < T0 in relation to NEC, first excluding all children receiving enemas before T0, and secondly excluding only the subjects with enema administration before the first passage of meconium.

## Results

**Fig. 1:**
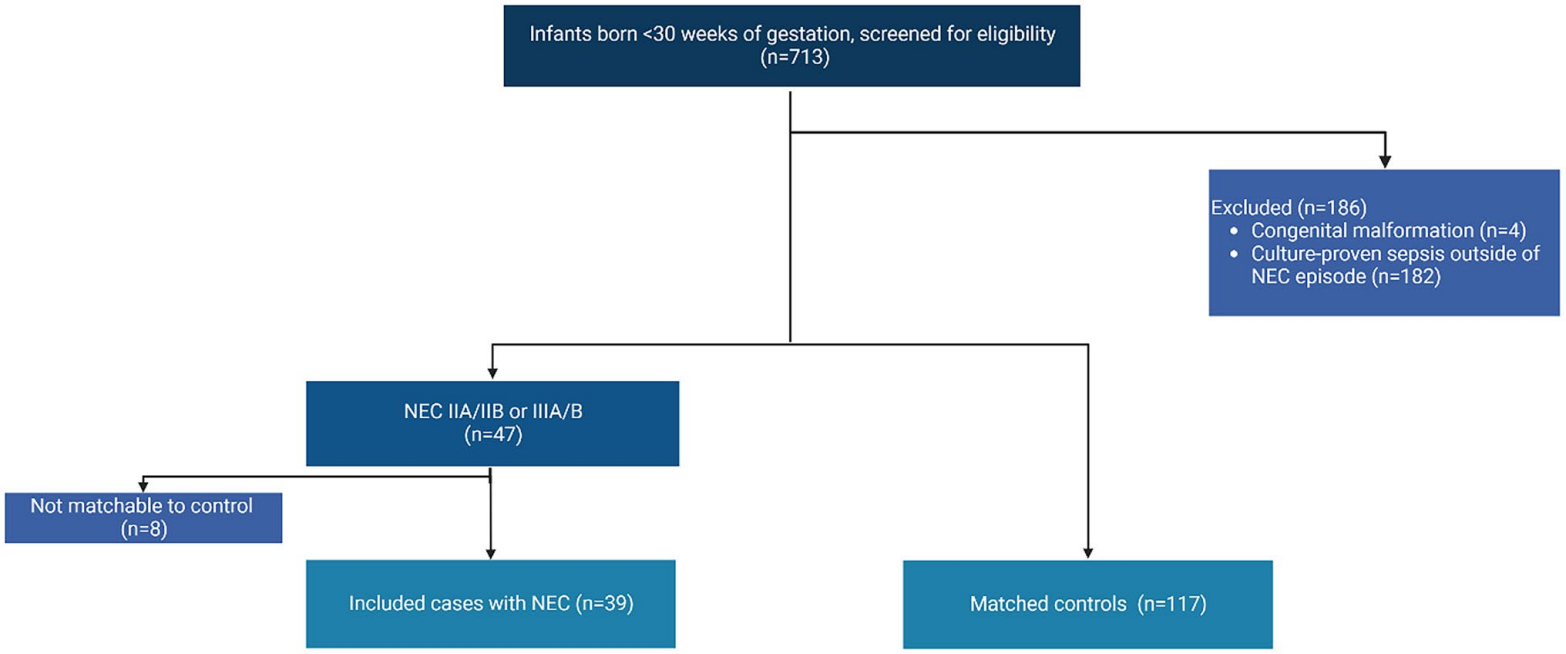
Flow chart of inclusion process (*NEC, necrotizing enterocolitis*)

Between December and January 2020, 713 preterm neonates were born at GA < 30 weeks. Forty-seven (7%) developed necrotizing enterocolitis, of which 39 could be 1:3 matched to 117 controls (Fig. 1). Gestational age ranged between 24 5/7 weeks and 29 6/7. Minimal enteral feeding was initiated on day 0 or 1 in 98% of cases and controls. Before T_0_, 10% of cases (n = 4) and 11% of controls (n = 13) were exposed to formula feeding for > 20% of the total enteral volume (Table 1).

**Table 1:** Baseline clinical and demographic characteristics

	NEC (n = 39)	Controls (n = 117)	p-value
Gestational age, *weeks* + *days,* median [IQR]	27 + 3 [26 + 1—28 + 6]	27 + 4 [26 + 0—29 + 0]	0.56
Gravidity mother, *first pregnancy*, n (%)	18 (46)	57 (49)	0.20
Gender, *female*, n (%)	12 (31)	60 (51)	0.03*
Singleton, n (%)	32 (82)	80 (60)	0.10
Pre-eclampsia mother, n (%)	6 (15)	23 (19)	0.57
Delivery mode, *caesarean section*, n (%)	21 (54)	58 (50)	0.64
Birth weight, *grams*, mean (SD)	978 (242)	967 (233)	0.81
SGA, n (%)	4 (10)	18 (15)	0.42
Apgar score 1 min, median [IQR]	6 [4—7]	5 [3—7]	0.36
Apgar score 5 min, median [IQR]	8 [6—8]	8 [7—8]	0.70
Surfactant administration, n (%)	25 (66)	70 (60)	0.55
Antibiotic administration < 24 h postnatal, n(%)	26 (67)	80 (68)	0.85
Invasive ventilation before T0^*a*^*,* n(%)	15 (39)	16 (14)	< 0.001*
Invasive ventilation before T0^*a*^, *days,* median [IQR]	0 [0—3]	0 [0—0]	0.001
Treatment for patent *ductus arteriosus*, n (%)	7 (18)	18 (15)	0.71
Erythrocyte transfusion before T0^a^, n(%)	17 (44)	36 (31)	0.14
Intraventricular hemorrhage gr. III or IV*,* n (%)	2 (5)	5 (4)	0.81
Minimal enteral feeding initiation (5–10 ml/kg/d)			0.89
* Day of life 0,* n (%)	32 (82)	94 (80)	
* Day of life 1,* n (%)	6 (15)	21 (18)	
* Day of life* ≥ *2,* n (%)	3 (2)	2 (2)	
Enteral feeding initiation (> 10 ml/day)			0.10
* Day of life 0 or 1,* n (%)	31 (80)	72 (62)	
* Day of life 2,* n (%)	5 (13)	34 (29)	
* Day of life* ≥ *3,* n (%)	3 (8)	11 (9)	
Age at full enteral feed	9 [8—11]	8 [7—10]	0.03*
Full enteral feeding before T0^a^, n (%)	29 (74)	94 (81)	0.37
> 20% average daily enteral volume consisting of formula milk before T0^*a*^, n (%)	4 (10)	13 (11)	0.83
Parenteral feeding before T0^*a*^, *days,* median [IQR]	8 [5—10]	6 [4—8]	0.01*
Enema administration before T0^*a*^, n (%)	14 (36)	35 (30)	0.55
Enema before first meconium passage, n (%)	5 (13)	18 (15)	0.51

Data are summarized as mean and standard deviation (SD) or number and percentage (%), unless stated otherwise

ml/kg/d milliliter per kilogram birth weight per day

NEC necrotizing enterocolitis, NICU neonatal intensive care unit, SGA small for gestational age

*p < 0.05

^a^T0 refers to the day of NEC diagnosis or the corresponding postnatal day of controls

One control was transferred before the corresponding postnatal age of onset of its matched case. DF < T0 was therefore based on 2 days, instead of 3 days. NEC Bell’s stage ≥ IIa was diagnosed at a median postnatal age of 12 days [IQR 8–18]. In 15 (38%) cases, surgical treatment for NEC was indicated based on clinical and radiographic signs. Three of these neonates deceased before surgery could be performed. All twelve surgically treated infants showed ileal or jejunal necrosis. Two of the surgically treated and five of the non-surgically treated infants died in the first 28 days of life. None of the controls deceased. Bloody stools were observed in 8% of cases before NEC diagnosis and in none of the controls. In almost half (45%) of the infants, NEC was associated with sepsis. No sepsis was observed in controls, in correspondence with the exclusion criteria. Cases were more often invasively ventilated before NEC onset than their peers in the control group (39% vs 14%; p < 0.001). Of all infants developing NEC, 74% reached full enteral feeds before T0 *vs.* 81% in the control group (p = 0.37) (Table 1). In the group that developed NEC, full enteral feeding was reached at day 9 [IQR 8–11] and in controls at day 8 [IQR 7–10] (p = 0.03) (Table 1). Parenteral feeding duration before T0 was significantly longer in cases *vs.* controls 8 [IQR 5—10] *vs.* 6 [IQR 4—8] days (p = 0.01) (Table 1).

Rectal enemas (saline) were administered in 49 (31%) infants before T_0_ (36% of cases *vs.* 30% of controls, p = 0.55). In 23 (15%) infants, enemas were administered before the first meconium passage. Of these 23 infants, the youngest was 48 h old, and the oldest was 6 days (median 3 days). Enema administration was highest in infants with TFPM > 72 h (82%), compared to infants with TFPM ≤ 24 h (16%), 24-48 h (19%), and 48-72 h (47%) (p = 0.001). In this cohort, no other interventions such as oral/rectal laxatives or abdominal/rectal stimulation were reported. Other demographic and clinical data are depicted in Table 1.

The TFPM ranged between 0 h and 7 days with a median of 36 and 30 h in cases and controls, resp. (p = 0.83) (Table 2). Only three infants (all controls) passed meconium beyond the first 120 h (5 days) of life. The proportion of infants with TFPM > 72 h did not differ between cases and controls (Table 3). A longer TFPM was positively correlated to duration of parenteral nutrition before T_0_, enema administration before passage of meconium, and the need for invasive ventilation (p = 0.02, p < 0.001, and p = 0.01, resp.). Gestational age was not correlated with TFPM in our cohort (p = 0.92), thus not corrected for in the multivariate regression analysis.

**Table 2:** Odds ratio for NEC by time to first passage of meconium, time between first passage of meconium and first transitional stool, and mean daily defecation

	NEC (median [IQR])	Controls (median [IQR])	Odds ratio [95%CI]	p-value	Adjusted odds ratio [95%CI]	p-value
Time to first meconium passage, *postnatal age in hours*	36 [13–65]	30 [9–66]	1.03 [0.82 – 1.28]	0.83	1.00 [0.99 – 1.03]^*a*^	0.41
Time between first passage of meconium and first transitional stool, *days*	4 [3–5]	4 [3–5]	1.04 [0.87 – 1.25]	0.65	1.16 [0.86 – 1.55]^*b*^	0.34
Mean daily defecation frequency in the three days preceding T0*, *median and IQR of mean*	3 [2–5]	3 [2–4]	0.99 [0.75 – 1.32]	0.96	0.97 [0.72–1.31]^*c*^	0.84

Data are summarized as odds ratio (95% confidence interval)

*CI* confidence interval, *NEC* necrotizing enterocolitis, *OR* odds ratio

*T0 refers to the day of NEC diagnosis or the corresponding postnatal day of controls

^a^Adjusted for need for invasive ventilation, length of parenteral nutrition before T0 (day of NEC onset) and enema administration before first meconium passage

^b^Adjusted for enema administration before first passage of meconium

^c^Adjusted for gestational age at birth

**Table 3:** Crosstab of time to first passage of meconium (categorical variable) with case/control status

	NEC (n = 39)	No NEC (n = 117)	p-value
TFPM < 24 h, n (%)	16 (41)	52(44)	0.86
TFPM 24-48 h, n (%)	76 (15)	21 (18)	
TFPM 48-72 h, n (%)	9 (23)	20 (17)	
TFPM > 72 h, n (%)	8 (21)	24 (21)	

*NEC* necrotizing enterocolitis, *TFPM* Time to first passage of meconium

Also, duration of meconial stool passage did not differ between cases and controls (4 *vs.* 4 days, p = 0.65) (Table 2). It was negatively correlated to enema administration before first passage of meconium (p < 0.001). Gestational age, invasive ventilation nor parenteral feeding duration before T_0_ were correlated to meconial passage duration (p = 0.95, p = 0.23, and p = 0.82, resp.).

Finally, DF < T0 did not differ between cases and controls (3 *vs.* 3 times daily, p = 0.71) (Table 2). DF < T0 ranged between 0 and 6 defecations daily and was inversely correlated to gestational age; for every extra week of gestation, DF < T0 decreased by 0.25 (p < 0.001). Parenteral feeding duration before T_0_, invasive ventilation, and enema administration were not correlated to DF < T0 (p = 0.30, p = 0.97, and p = 0.61, resp.).

NEC was not associated with TFPM, number of days of meconium passage, nor DF < T0 when excluding children receiving enemas before T0, nor when excluding only the subjects with enema administration before the first passage of meconium (Supplementary Table S1).

## Discussion

We assessed three different parameters of gut motility (TFPM, duration of meconium passage until transitional stool, and DF < T0) in association with NEC in preterm infants (GA < 30 weeks). In this case–control study, there was no association between these defecation parameters and the development of NEC.

Firstly, TFPM did not differ between infants developing NEC compared to matched controls, when analyzed as both a categorical and continuous variable. In both cases and controls, about 40% passed meconium within 24 h after birth and about 40% after 48 h, which is comparable to other preterm cohorts [24, 25]. This is in line with the results of Gregory et al. from a large cohort (129 NEC *vs.* 129 controls), where the postnatal day of first meconium passage was comparable between controls and infants developing NEC Bell’s stage ≥ IIa [19]. In comparison, Andrews and Kworchuks showed a trend towards an increased TFPM (in hours) in infants developing NEC Bell’s stage ≥ IIa in a sample of 34 cases and 34 controls (mean TFPM 43.1 *vs.* 34.7, p = 0.08) [18]. However, the precision of time registration differed between studies. Andrews et al. reported to have assessed TFPM per hour (up to one decimal), whilst in our study, we collected data per 3 h, and Gregory et al. collected data per 24 h. In real life, the production of stools is not continuously assessed in the NICU setting, but only during nurse care moments when the infant is not asleep. The exact moment of stool production is therefore difficult to evaluate and this type of data always carries a certain inaccuracy.

Additional factors influencing TFPM and complicating comparisons between studies include interventions to stimulate defecation. Neither Andrews et al*.* nor Gregory et al*.* report on such interventions. In our cohort, no laxatives were used, but saline enemas were administered in about 1/3 of cases and controls (30 and 36%, resp.). In our centers, enemas are often administered in case a neonate has not passed meconium within 48 h after birth, or within 24 h after birth if there are mild to moderate gastrointestinal symptoms, such as gastric retention and abdominal distension, outside of the suspicion of NEC. The decision to administer rectal saline is healthcare provider-dependent, explaining why not all infants with TFPM > 48 h were treated with rectal saline. In our cohort, enemas were administered widely, which is likely to have expedited TFPM in neonates receiving enemas [26]. After excluding neonates to whom enemas had been administered, TFMP remained comparable between cases and controls, suggesting enema administration did not meaningfully confound our results.

Secondly, the duration of meconium production was comparable in cases and controls. The duration of meconium production is inversely correlated with gestational age, suggesting developmental age influences the transition to normal stools [24, 27]. As NEC is also associated with immaturity, we had expected an increased meconium production duration in NEC cases compared to controls. Except for the relation with immaturity, there is no available literature on the correlation of NEC with duration of meconium production. This might be a parameter worth exploring in future studies.

Thirdly, we did not observe differences in DF < T0 between cases and controls. The association between defecation frequency and the development of NEC has only scarcely been studied [18, 19, 28]. In accordance with our results, Gregory et al*.* did not find significant differences in defecation frequency in the three days preceding NEC, compared to controls. However, when categorizing cases according to Bell’s staging, NEC IIa-b was associated with a higher number of maximum and mean stools per day in the first week of life, but not in weeks 2–4 [19]. Andrews and Kworchuks evaluated defecation frequency in preterm infants developing NEC on three separate days (on days 6, 2, and 1 prior to NEC onset) and compared this with the defecation frequency of controls at the corresponding postnatal age [18]. The authors reported a higher defecation frequency in cases *vs.* controls on each of these three days preceding NEC [18]. Interestingly, a recently published animal study partially corroborated these findings [28]. In the latter study, gastrointestinal transit times (assessed via abdominal X-rays after ingestion of contrast solution) were studied in preterm piglets with macroscopic NEC lesions. The authors found differences in transit times between piglets with NEC lesions in the small intestine versus NEC lesions in the colon. NEC lesions in the small intestine were associated with delayed transit time in both the small and large bowel, while colonic lesions were associated with accelerated small bowel transit times and diarrhea. It is unclear whether the localization of NEC lesions in preterm infants would influence the transit time in the same manner as described in piglets [28]. Since none of the surgically-treated infants from our cohort showed colonic involvement, an analysis of transit times in case of colonic versus small intestine lesions could not be performed.

Based on our and previous results, there is currently not enough evidence that risk assessment for NEC is possible by analyzing defecation patterns as assessed in this study. In the future, other characteristics, such as changes in defecation frequency and fecal aspect should be studied in this regard. Potential confounders in stool evaluation should also be kept in mind. It should be brought to attention that enteral feeding type and parenteral feeding volume can be associated with both defecation patterns and NEC [29, 30]. In our cohort, most infants predominantly received human milk, without differences between cases and controls. Parenteral feeding was, however, more common and prolonged in cases before T_0_ and was, therefore, considered a potential confounder in the multivariate analysis. Whether a child was already on *nil per os* regimen before the diagnosis of NEC Bell’s stage ≥ IIa, was unknown in this study. This should also be considered in future studies where defecation parameters are investigated. Also invasive mechanical ventilation was considered as confounder, as it was more common in the case group. This could be associated with decreased gastrointestinal motility due to opiate administration [31].

The current study adds to the scarce literature on defecation patterns in preterm infants in relation to the development of NEC. Strengths of our study include the diligent matching process and the detailed, standardized data collection which enables identification of potential confounders. As insights into NEC pathophysiology and symptomatology have progressed since the above-mentioned previously published studies, our cohort is likely to consist of fewer NEC-resembling diagnoses, such as milk-curd syndrome and SIP, as described in the exclusion criteria [32]. This smaller but more homogeneous cohort could be considered more informative than the cohort previously described by Gregory et al., with 42 of 129 NEC cases being staged as NEC I, and thus not confirmed NEC [19].

Our study also has several limitations. Despite the case–control design, feeding, and ventilation type were different in both groups. Although this was corrected for in the statistical analysis, there might be a residual effect on the result. Secondly, the collection of data on defecation patterns was performed retrospectively, as an addition to the prospective data collection of the original study. Because no standardized scoring system for defecation was used, the volume and consistency of feces were not assessed in this study. Finally, the aspect of transitional stools was based on the non-standardized nurse reports. Furthermore, as the number of cases developing severe NEC was relatively small, it was not possible to analyze defecation patterns across the different Bell’s stages.

In conclusion, although the development of NEC is closely linked to factors related to the immaturity of the gut, in this study the development of NEC was not associated with signs of dysmotility such as TFPM, duration of meconium passage, or DF < T0. These potential signs of gut dysmotility should not be considered as early warning signs for NEC based on our results. It remains to be elucidated whether the location of affected gut segments and the staging of NEC are associated with differences in bowel habits in preterm neonates.

### Supplementary Information

Below is the link to the electronic supplementary material.

Supplementary file1 (DOCX 15 KB)

## Data Availability

The data used in the current study are available upon reasonable request.

## Abbreviations

aOR: Adjusted odds ratio
CI: Confidence interval
DF < T0: Mean defecation frequency per day over the 72 h preceding clinical NEC onset
ENS: Enteric nervous system
FM: Formula milk
HM: Human milk (mother’s own milk or donor milk)
IQR: Interquartile range
LOS: Late-onset sepsis
NEC: Necrotizing enterocolitis
NICU: Neonatal intensive care unit
OR: Odds ratio
SD: Standard deviation
SPSS: Statistical Package for Social Sciences
T_0_: Day of diagnosis of necrotizing enterocolitis or corresponding postnatal day in controls
TFPM: Time to first passage of meconium
