# Supplementary material for: Time to first passage of meconium and defecation frequency preceding necrotizing enterocolitis in preterm infants: a case–control study
Source: Eur J Pediatr. 2023 Jun 22;182(9):3907–15. doi: 10.1007/s00431-023-05035-8 (PMC10570237; doi:10.1007/s00431-023-05035-8)
Supplement: Supplementary file 1 — Supplementary file1 (DOCX 15 KB) [file 431_2023_5035_MOESM1_ESM.docx]

| Table S1. Odds ratio NEC by time to first passage of meconium, time between first passage of meconium and first transitional stool, and mean daily defecation (infants who received a rectal enema before first passage of meconium and before T0 excluded). | | | | | | | |
| --- | --- | --- | --- | --- | --- | --- | --- |
|  | NEC (median [IQR]) | Controls (median [IQR]) | Odds ratio [95%CI] | p – value | Adjusted odds ratio [95%CI] | p – value |  |
| Excluding infants who received a rectal enema before T0* (n=107) | | | | | | |  |
| Time to first meconium passage, *postnatal age in hours* | 19 [6 – 14] | 23 [8 – 46] | 1.00 [0.98 – 1.02] | 0.96 | 1.00 [0.98 – 1.02]^a^ | 0.80 |  |
| Time between first passage of meconium and first transitional stool, *days* | 4 [3 – 5] | 4 [3 – 5] | 0.97 [0.74 – 1.26] | 0.79 | NA | NA |  |
| Mean daily defecation frequency in the three days preceding T0*, *median and IQR of mean* | 3 [3 – 5] | 3 [2 – 4] | 1.05 [0.75 – 1.47] | 0.77 | 1.00 [0.70 – 1.42]^b^ | 0.99 |  |
| Excluding infants who received a rectal enema before the first passage of meconium (n=134) | | | | | | |  |
| Time to first meconium passage, *postnatal age in hours* | 26 [11 – 57] | 23 [7 – 53] | 1.00 [0.99 – 1.02] | 0.60 | 1.00 [0.99 – 1.01]*^a^* | 0.80 |  |
| Time between first passage of meconium and first transitional stool, *days* | 4 [3 – 5] | 4 [2 – 4] | 1.03 [0.84 – 1.25] | 0.81 | 1.00 [0.81 – 1.24]*^c^* | 0.99 |  |
| Mean daily defecation frequency in the three days preceding T0*, *median and IQR of mean* | 3 [2 – 4] | 3 [2 – 5] | 1.07 [0.79 – 1.45] | 0.65 | 1.08 [0.80 – 1.47]*^b^* | 0.62 |  |
| **T0 refers to the day of NEC diagnosis or the corresponding postnatal day of controls.*  *^a^Adjusted for need for invasive ventilation, length of parenteral nutrition before T0 (day of NEC onset) and enema administration before first meconium passage.*  *^b^ Adjusted for gestational age at birth*  *^c^Adjusted for enema administration before T0*  *CI, confidence interval; NEC: necrotizing enterocolitis; OR: odds ratio. Data are summarized as odds ratio (95% confidence interval).* | | | | | | | |
